# Non-invasively Measured Venous Oxygen Saturation as Early Marker of Impaired Oxygen Delivery in Preterm Neonates

**DOI:** 10.3389/fped.2022.834045

**Published:** 2022-01-28

**Authors:** Lukas P. Mileder, Julia Buchmayer, Nariae Baik-Schneditz, Bernhard Schwaberger, Nina Höller, Chad C. Andersen, Michael J. Stark, Gerhard Pichler, Berndt Urlesberger

**Affiliations:** ^1^Research Unit for Neonatal Micro- and Macrocirculation, Department of Pediatrics and Adolescent Medicine, Medical University of Graz, Graz, Austria; ^2^Division of Neonatology, Department of Pediatrics and Adolescent Medicine, Medical University of Graz, Graz, Austria; ^3^Division of Neonatology, Intensive Care and Neuropediatrics, Department of Pediatrics and Adolescent Medicine, Comprehensive Center for Pediatrics, Medical University of Vienna, Vienna, Austria; ^4^Department of Neonatal Medicine, Women's and Children's Hospital, North Adelaide, SA, Australia; ^5^School of Medicine, Robinson Research Institute, University of Adelaide, Adelaide, SA, Australia

**Keywords:** preterm neonate, inflammation, infection, near-infrared spectroscopy, tissue oxygenation, venous oxygen saturation

## Abstract

**Introduction:**

Adequate oxygen supply for preterm neonates may be defined through non-invasive measurement of venous oxygen saturation (SvO_2_) and fractional oxygen extraction using near-infrared spectroscopy (NIRS). We investigated whether there was a difference in peripheral muscle SvO_2_ (pSvO_2_) and peripheral fractional oxygen extraction (pFOE) in preterm neonates with early inflammation/infection compared to healthy subjects during the first 72 h after birth.

**Materials and Methods:**

We retrospectively analyzed secondary outcome parameters of prospective observational studies, including preterm neonates at risk of infection in whom peripheral NIRS measurements were performed in combination with venous occlusions. Early neonatal inflammation/infection was diagnosed by clinical signs and laboratory parameters. Peripheral muscle tissue oxygenation index (pTOI) was measured using either NIRO 300 or NIRO 200-NX (both Hamamatsu Photonics, Japan) on the patients' lower legs. Using 20-s venous occlusions, pSvO_2_ and pFOE were calculated incorporating simultaneous measurements of arterial oxygen saturation (SpO_2_).

**Results:**

We analyzed measurements from 226 preterm neonates (median gestational age 33.9 weeks), 64 (28.3%) of whom were diagnosed with early neonatal inflammation/infection. During the first 24 h after birth, pSvO_2_ (66.9% [62.6–69.2] vs. 69.4% [64.6–72.0]; *p* = 0.04) and pTOI (68.6% [65.3–71.9] vs. 71.7% [67.3–75.1]; *p* = 0.02) were lower in those neonates with inflammation/infection, while there was no such difference for measurements between 24–48 and 48–72 h.

**Discussion:**

NIRS measurement of pSvO_2_ and pFOE is feasible and may be utilized for early detection of impaired peripheral oxygen delivery. As pTOI was also significantly lower, this parameter may serve as substitute for diminished regional oxygen supply.

## Introduction

Adequate assessment of patients' oxygenation status and the estimation of their oxygen demand are challenging, especially in preterm neonates. Changes in arterial oxygen saturation (SpO_2_) occur relatively late during oxygen deficiency and, therefore, methods to detect an imbalance between oxygen demand and supply earlier would have the potential to decrease neonatal morbidity and mortality (1, 2).

To define “adequacy of oxygenation” in preterm neonates it has been recently suggested by Andersen et al. (3, 4) to focus on the venous oxygen reservoir rather than on SpO_2_, as the latter may be relatively constant even in times of low oxygen supply or increased oxygen consumption (3). Venous oxygen saturation (SvO_2_) depends on SpO_2_, hemoglobin fractions and levels (i.e., oxygen carrying capacity), cardiac output, and tissue oxygen consumption (VO_2_) according to Fick's equation (5). While invasive measurement of SvO_2_ is impractical in preterm neonates, utilization of near-infrared spectroscopy (NIRS) offers the possibility to measure SvO_2_ from peripheral muscle tissue non-invasively.

Following the publications of Andersen et al. (3, 4) we approached the question of adequacy of oxygenation with an analysis of SvO_2_ in preterm neonates by using NIRS. Adequacy implies a sufficient quantity of oxygen to satisfy tissue needs, thus in preterm neonates we assessed the amount of excess oxygen left over after all the processes of cellular respiration were completed as a proof of concept (3). We hypothesized to see differences in VO_2_ and oxygen delivery (DO_2_) due to impaired microcirculation in early neonatal inflammation/infection. With impairment of oxygen delivery likely being a dynamic process, we took changes over time into account and, thus, divided the observation period of 72 h after birth into three 24-h intervals.

## Materials and Methods

For this study, we retrospectively analyzed secondary outcome parameters of prospective observational studies that were performed between November 30^th^, 2005, and June 28^th^, 2015, at the Division of Neonatology, Medical University of Graz, Austria. We included preterm neonates at risk of infection in whom NIRS measurements of peripheral muscle tissue had been performed in combination with venous occlusions during the first 72 h after birth. Risk factors for early neonatal inflammation/infection included premature rupture of membranes, chorioamnionitis, and vaginal colonization with group B streptococcus, among others. Exclusion criteria were congenital cardiovascular malformations and perinatal asphyxia. All studies had been approved by our university's ethics committee (ethics committee numbers 19-291 ex 07/08, 21-149 ex 09/10, 23-402 ex 10/11, and 25-237 ex 12/13) and written parental consent had been secured prior to study inclusion.

We obtained demographic and clinical data, including gestational age, birth weight, sex, umbilical artery pH, and Apgar scores from clinical records. Neonates were differentiated by the development of early neonatal inflammation/infection. For this purpose, a blood culture was taken on the first day of life (either from umbilical cord or infants' blood); according to previously published criteria (6), early inflammation/infection was defined by characteristic clinical signs and a positive blood culture and/or a C-reactive protein (CrP) level above the cut-off value of 10 mg/l. Patient were stratified by chronological age at the time of NIRS measurements into three sub-groups: measurement within 24 h after birth, between 24 and 48 h after birth, and between 48 and 72 h after birth, respectively.

### NIRS Measurements

According to previously published protocols (7), NIRS measurements were performed under standardized conditions during undisturbed daytime sleep. Neonates were placed in supine position and tilted up (10°), while their legs were positioned just above mid-sternum level. We used either the NIRO 300 or NIRO 200-NX (both Hamamatsu Photonics, Hamamatsu City, Japan) to measure peripheral muscle tissue oxygenation, with appropriately sized NIRS probes being placed on the patients' calves. For venous occlusions, a blood pressure cuff was placed around the ipsilateral thigh and inflated to a pressure level between venous and diastolic arterial pressure, thus interrupting venous outflow but leaving arterial inflow undisturbed. Thus, changes in total, oxygenated and deoxygenated hemoglobin during venous occlusions were only caused by arterial inflow and oxygen consumption of tissue, ensuring reliable measurements. The cuff was kept inflated for 20 s and NIRS data were recorded. SpO_2_ [%] and heart rate (HR [beats/min]) were measured with a pulse oximeter on the ipsilateral foot (IntelliVue MP30, Philips, The Netherlands). Occlusions were repeated until at least one measurement passed the quality criteria as defined by Pichler et al. (8).

This procedure allowed measurement of total, oxygenated and deoxygenated hemoglobin as well as peripheral muscle tissue oxygenation index (pTOI), while peripheral venous oxygen saturation (pSvO_2_) [%], peripheral fractional oxygen extraction (pFOE) [%], DO_2_ [μmol/l/min], and VO_2_ [μmol/l/min] were calculated from the abovementioned parameters through integration of SpO_2_ data (8).

### Statistical Analysis

Statistical analyses were performed using IBM SPSS Statistics 25 (Armonk, United States of America). Metrically scaled data were tested for normal distribution using the Kolmogorov-Smirnov-test. Normally distributed data are presented as mean ± one standard deviation, while skewed data are presented as median and interquartile range [25^th^ and 75^th^ percentile]. For calculation of group differences, we used Student's *t-*test, Chi-square test or Mann-Whitney-*U*-test, as appropriate. A two-sided *p* < 0.05 was considered as statistically significant.

## Result

During the study period, NIRS measurements with venous occlusions were performed in 499 neonates. A total of 273 patients were excluded due to several reasons: term neonates (*n* = 113), measurements later than 72 h after birth and/or missing data (*n* = 109), perinatal asphyxia (*n* = 33), and different measurement method (*n* = 18). Hence, NIRS measurements from 226 preterm neonates with a median gestational age of 33.9 (32.4–35.0) weeks were analyzed.

**Figure 1:**
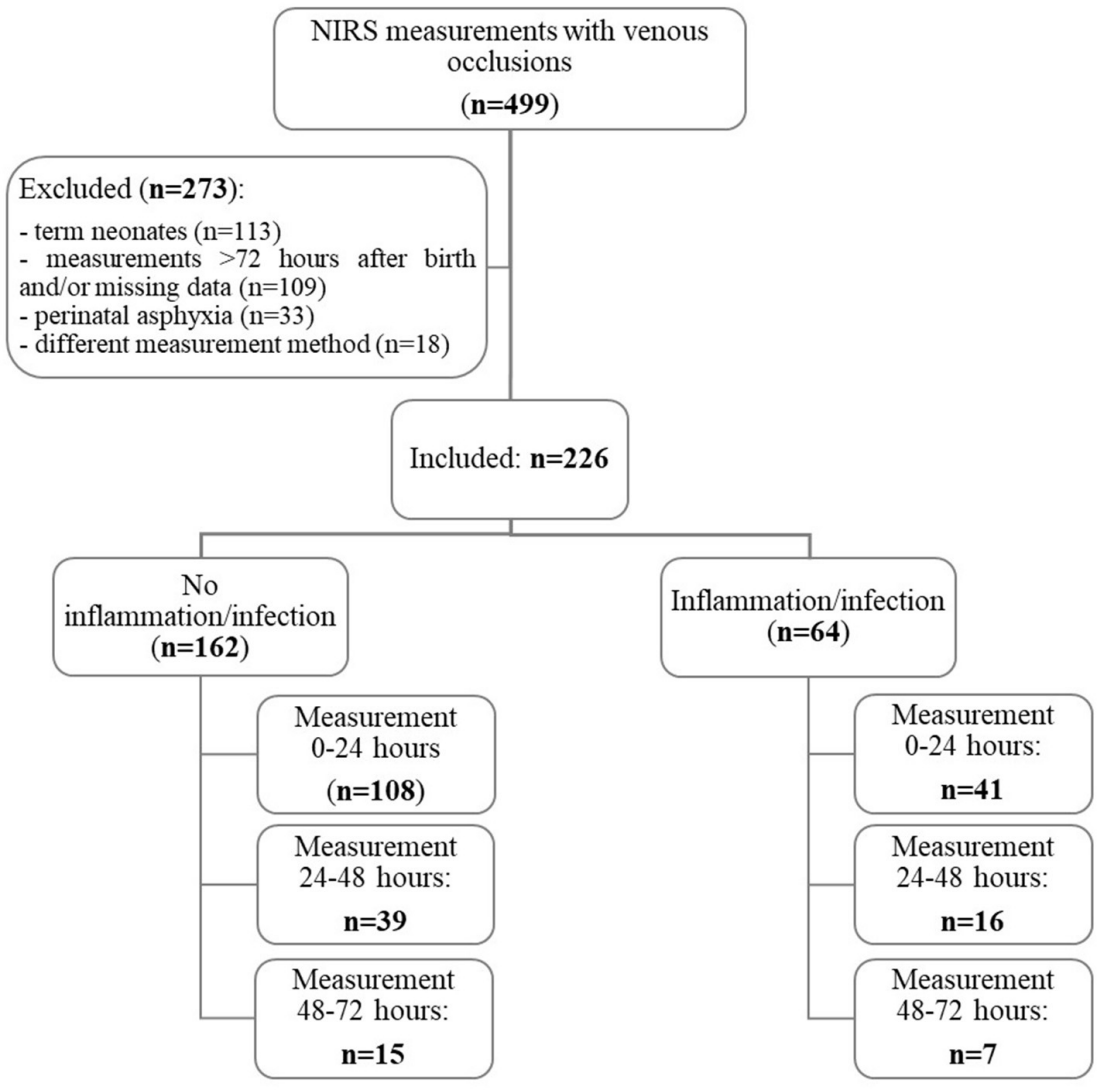
Patient flow diagram.

Of the 226 included patients, 64 (28.3%) were diagnosed with early neonatal inflammation/infection. There were no relevant demographic and clinical differences between patients in the non-inflammation/infection and those in the inflammation/infection group (Table 1). In the inflammation/infection group (*n* = 64), 41 measurements (64.1%) were performed within 24 h after birth, 16 (25%) between 24 and 48 h, and seven (10.9%) between 48 and 72 h. In the non-inflammation/infection group (*n* = 162), 108 measurements (66.7%) were performed within 24 h after birth, 39 (24.1%) between 24 and 48 h, and 15 (9.3%) between 48 and 72 h (Figure 1).

**Table 1:** Demographic and clinical data of healthy preterm neonates and those with early inflammation/infection: median and interquartile range [25^th^ and 75^th^ percentile] or relative frequency.

	**No inflammation/infection (*n* = 162)**	**Inflammation/infection (*n* = 64)**	***p*-value**
Gestational age (weeks)	34.0 [32.4–35.0]	33.7 [32.0–35.3]	0.61
Birth weight (g)	2,095 [1,698–2,449]	2,115 [1,717–2,565]	0.86
Male sex	100 [61.7%]	42 [65.6%]	0.59
Umbilical artery pH	7.30 [7.24–7.34]	7.31 [7.22–7.33]	0.99
Apgar 1 min	8 (8, 9)	8 (8, 9)	0.59
Apgar 5 min	9 (9, 10)	9 (8–10)	0.63
Apgar 10 min	9 (9, 10)	9 (9, 10)	0.99
Catecholamine/ inotropic support during 72 h after birth	9 [5.6%]	4 [6.3%]	0.84

In the sub-group with NIRS measurements during the first 24 h after birth (*n* = 149), both pSvO_2_ [66.9% (62.6–69.2) vs. 69.4% (64.6–72.0); *p* = 0.04] and pTOI [68.6% (65.3–71.9) vs. 71.7% (67.3–75.1); *p* = 0.02) were significantly lower in the inflammation/infection group compared to the non-inflammation/infection group. DO_2_ was lower in the inflammation/infection group, but this difference did not reach statistical significance (*p* = 0.13). pFOE was higher in the inflammation/infection group, but this difference did not reach significance either (*p* = 0.15). All other parameters (VO_2_, SpO_2_, and HR) did not differ between the two groups (Table 2).

**Table 2:** Outcome parameters presented as median and interquartile range [25^th^ and 75^th^ percentile] compared between healthy preterm neonates and those with early inflammation/infection during the first 72 h after birth.

**0–24 h**	**No inflammation/infection (*n* = 108)**	**Inflammation/infection (*n* = 41)**	***p*-value**
pSvO_2_ (%)	69.4 [64.6–72.0]	66.9 [62.6–69.2]	0.04*
pFOE (%)	28.0 [24.3–32.9]	30.1 [26.9–33.9]	0.15
pTOI (%)	71.7 [67.3–75.1]	68.6 [65.3–71.9]	0.02*
DO_2_ (μmol/l/min)	44.7 [28.8–62.4]	34.7 [27.0–58.3]	0.13
VO_2_ (μmol/l/min)	12.5 [8.7–15.8]	11.2 [8.0–14.9]	0.35
SpO_2_ (%)	96.7 [93.7–98.3]	95.7 [93.0–97.6]	0.35
HR (bpm)	131 [123–141]	134 [123–149]	0.49
**24**–**48 h**	**No inflammation/** **infection** **(*****n*** **=** **39)**	**Inflammation/** **infection** **(*****n*** **=** **16)**	***p*** **-value**
pSvO_2_ (%)	70.0 [65.0–74.0]	67.5 [65.3–71.9]	0.45
pFOE (%)	27.0 [23.0–33.0]	29.1 [22.0–31.5]	0.70
pTOI (%)	73.2 [67.9–75.7]	69.8 [67.7–73.5]	0.17
DO_2_ (μmol/l/min)	51.9 [32.0–66.7]	39.8 [24.7–49.4]	0.77
VO_2_ (μmol/l/min)	12.8 [9.7–16.2]	10.4 [7.9–13.2]	0.23
SpO_2_ (%)	96.7 [95.2–98.4]	93.4 [91.8–96.5]	0.00*
HR (bpm)	127 [117–135]	127 [119–138]	0.81
**48**–**72 h**	**No inflammation/** **infection** **(*****n*** **=** **15)**	**Inflammation/** **infection** **(*****n*** **=** **7)**	***p*** **-value**
pSvO_2_ (%)	68.0 [62.0–72.0]	71.0 [64.0–74.0]	0.47
pFOE (%)	28.0 [25.0–35.0]	26.0 [23.0–32.0]	0.63
pTOI (%)	69.2 [66.2–73.9]	74.0 [67.4–75.7]	0.40
DO_2_ (μmol/l/min)	38.3 [32.2–57.2]	50.9 [37.1–78.3]	0.33
VO_2_ (μmol/l/min)	12.3 [9.2–15.8]	13.1 [11.1–18.1]	0.55
SpO_2_ (%)	96.6 [93.4–99.0]	95.6 [91.7–97.1]	0.90
HR (bpm)	137 [125–147]	126 [123–134]	0.41

pSvO_2_, peripheral muscle venous oxygen saturation; pFOE, peripheral muscle fractional oxygen extraction; pTOI, peripheral muscle tissue oxygenation index; DO_2_, oxygen distribution; VO_2_, oxygen consumption; SpO_2_, arterial oxygen saturation; HR, heart rate; bpm: beats per minute; *p <0.05.

Between 24 and 48 h after birth (*n* = 55 measurements), SpO_2_ values were significantly lower in the inflammation/infection group [93.4% (91.8–96.5) vs. 96.7% (95.2–98.4); *p* = 0.001], while pSvO_2_, pFOE, pTOI, DO_2_, VO_2_, and HR were similar between the groups.

Between 48 and 72 h after birth (*n* = 22 measurements), we did not find any differences in pSvO_2_, pFOE, pTOI, DO_2_, VO_2_, SpO_2_, and HR between neonates in the inflammation/infection group and those without inflammation/infection.

## Discussion

The aim of the present study was to provide proof of concept that it is possible to determine adequacy of oxygenation through NIRS derived measurement of pSvO_2_. Further, differences in pSvO_2_, as a result of altered VO_2_ and DO_2_ due to impairment of the microcirculation, would be evident in early neonatal inflammation/infection. First, our study showed that calculation of pSvO_2_ and pFOE using non-invasive NIRS measurements is feasible in preterm neonates and may contribute relevant information to inform clinical practice. Second, within the first 24 h after birth, we did see significantly lower pSvO_2_ values in preterm neonates with early neonatal inflammation/infection, suggesting a reduced state of oxygen redundancy despite normal SpO_2_ values. Third, within the same period, we found significantly lower pTOI values in those neonates.

Within the first 24 h after birth there were lower DO_2_ values and higher pFOE values in the inflammation/infection group, but these differences did not reach statistical significance. Nevertheless, we view this observation as important, as reduced DO_2_ and higher pFOE would explain the significantly lower pSvO_2_ and pTOI values in the inflammation/infection group. Correspondingly, Pichler et al. (9) found decreased DO_2_ and pSvO_2_ in clinically stable neonates with elevated CrP during the first week after birth. Barcroft was the first to classify three key determinants of reduced oxygen delivery: stagnant hypoxia (from low blood flow), anemic hypoxia (from low hemoglobin) or hypoxic hypoxia (from low inspired oxygen tension) (3). We strongly consider predominantly a situation of stagnant hypoxia (diminished local blood flow) being responsible for our observation. We cannot rule out anemic hypoxia, as we did not measure hemoglobin directly, but there is no reason to assume significant differences between both groups. Hypoxic hypoxia is improbable, given that there were no differences in SpO_2_ between the groups. In an early phase of inflammation/infection, a reduction in peripheral blood flow due to local changes in perfusion seems very appropriate from a pathophysiological point of view. This assumption fits well to the increase in pFOE to compensate for the local reduction in DO_2_.

It is interesting to note that our groups did not differ in VO_2_, while Mrozek et al. (10) reported elevated oxygen consumption with increasing degree of neonatal sepsis. VO_2_ was not elevated in our inflammation/infection group at all. We consider this observation as a further hint at a reduction in local blood flow, as VO_2_ is defined as blood flow times oxygen extraction. As there was an increase in pFOE, but no change inVO_2_, we believe that the main reason for that observation was a reduction in local blood flow. However, all the changes were not very distinct, corresponding to Mrozek's categorization of “sick-non-septic,” as all neonates were in a stable clinical condition (10). All neonates received antibiotic treatment within the first 24 h and it seems therefore not surprising to us that we did not see any of these changes in the later course of the neonates.

The reduced pTOI values in the inflammation/infection group during the first 24 h and thereafter display the situation defined above perfectly. As pTOI reflects the balance of local DO_2_ and VO_2_, a reduction of pTOI would be expected. From a clinical perspective this reduction may be interpreted as a sign of centralization of blood flow (11). Similarly, a prospective observational study found impaired peripheral muscle tissue oxygen saturation during the first week of life in term and preterm neonates with CrP levels above 10 mg/l in comparison to healthy controls in a situation of cardio-circulatory stability (9). This emphasizes the potential of NIRS measurements to identify situations of diminished local DO_2_, which is an information of potential clinical relevance.

Studies from other disciplines such as cardiac surgery and adult intensive medical care underline the vital importance of a normoxic SvO_2_ for different medical conditions. Svenmarker et al. (12) reported significantly reduced patient survival at 30 days and 3 years after cardiac surgery to be associated with a central SvO_2_ below 75% during cardiopulmonary bypass. In the study by Boulain et al. (13) an initial central SvO_2_ below 70% was strongly associated with 28-day mortality in patients suffering from septic shock. Using serial hemodynamic measurements from pulmonary artery catheters in septic patients, Heiselman et al. (14) described a mixed SvO_2_ of <65% as “clinically unacceptable” and to be associated with poor prognosis. Correspondingly, in their case series of invasive SvO_2_ measurements in three neonates, Iwashima et al. (15) reported on a preterm neonate with an initial central SvO_2_ of 65% that ultimately died 10 days after birth due to renal failure. Although these invasively measured data cannot be directly compared with our results, it is relevant to note that preterm neonates with early neonatal inflammation/infection had a low mean pSvO_2_ of 65.9 ± 6.3% during the first day after birth in our study.

There is also a limited number of studies addressing SvO_2_ in the pediatric population. In children and adolescents with severe sepsis or fluid-refractory septic shock, de Oliveira et al. (16) showed a reduction in 28-day-mortality from 39.2 to 11.8% when aiming for a central SvO_2_ above 70%. Another study found significantly decreased in-hospital mortality and a lower number of dysfunctional organs in those children suffering from fluid-refractory septic shock whose therapy had been guided by intermittent central SvO_2_ monitoring (17). While it may be difficult to directly compare peripherally measured SvO_2_ with values derived from invasive measurements with the tip of a catheter at the junction of the inferior or superior vena cava and the right atrium, we found pSvO_2_ values close to 70%—which had been used as a target value in the two abovementioned studies—in the non-inflammation/infection group.

The most common alternative for SvO_2_ monitoring, i.e., placement of a central venous or a pulmonary artery catheter, is associated with relevant risks in neonates (18, 19), requires skill and expertise to be established, and often is limited by preterm neonates' body size. SvO_2_ measured from peripheral blood samples showed a rather good correlation with central SvO_2_ in adult patients undergoing cardiac surgery (20). More importantly, Samraj et al. (21) described a significant positive correlation between NIRS measurements of thenar muscle tissue oxygen saturation and central SvO_2_ in children suffering from shock. This finding indicates that non-invasively measured SvO_2_ from peripheral muscle tissue may be used as a valid surrogate for central SvO_2_.

Several clinical applications of NIRS-based measurements have already been described in neonatology. A prospective randomized controlled pilot trial found that supplemental oxygen delivery guided by cerebral tissue oxygenation monitoring reduced the burden of cerebral hypoxia in preterm neonates during the first 15 min after birth (22). Furthermore, simultaneous monitoring of cerebral and peripheral tissue oxygenation index combined with targeted interventions to treat arterial hypotension led to a non-significant reduction in the number of hypotensive episodes in preterm neonates (6). Our analysis was planned as a proof-of-concept study and, therefore, we are hesitant to define specific clinical applications of our findings yet. However, given our preliminary evidence and review of current literature, a pSvO_2_ below 65% should be considered as a state of impaired oxygenation in preterm neonates during the first day after birth.

### Limitations

This was a retrospective data analysis with all its inherent limitations. Further, we did not differentiate between various bacteria (e.g., Gram-positive and Gram-negative bacteria), which may potentially cause different cardiovascular effects. Still, our findings may be used as a hypothesis generating starting point for a prospective study with predefined clinical outcome parameters and an adequately sized patient cohort, investigating the additional benefit of pSvO_2_ monitoring for the definition of adequate oxygenation in preterm neonates.

When interpreting non-invasively measured pSvO_2_ it has to be taken into account that NIRS derived data is influenced by several demographic (e.g., gestational age, birth weight, calf diameter) and clinical parameters (HR, carbon dioxide partial pressure, peripheral body temperature, hemoglobin concentration, lactate) (23). Being fully aware of these associations, we were not able to control for these parameters given the retrospective nature of our analysis. Yet, we stringently used published quality criteria (8) to decrease test-retest variability and to improve reproducibility of peripheral NIRS measurements.

## Conclusion

pSvO_2_ was significantly reduced during the first 24 h after birth in preterm neonates of the inflammation/infection group, while SpO_2_ was similar between groups during this period. As pTOI was reduced at the same time, this parameter may be used as a proxy for these changes in clinical settings.

More research is certainly required to determine reference values for pSvO_2_ in preterm neonates and to investigate the safety and clinical potential of pSvO_2_ guided therapies. Yet, our preliminary findings suggest that NIRS measured pSvO_2_ and pTOI may be a promising non-invasive tool to detect neonates “at risk” early and to define oxygen sufficiency in preterm neonates.

## Data Availability Statement

The raw data supporting the conclusions of this article will be made available by the authors upon reasonable request.

## Ethics Statement

The studies involving human participants were reviewed and approved by the Ethics Committee, Medical University of Graz, Austria (19-291 ex 07/08, 21-149 ex 09/10, 23-402 ex 10/11, and 25-237 ex 12/13). Written informed consent to participate in this study was provided by the participants' legal guardian/next of kin.

## Author Contributions

LM and BU conceptualized the study, analyzed data, drafted the initial manuscript, and revised the manuscript. JB designed the data collection instruments, collected data, carried out the initial analyses, and critically reviewed the manuscript for important intellectual content. NB-S, BS, NH, and GP collected data, coordinated and supervised data collection, and critically reviewed the manuscript for important intellectual content. CA and MS conceptualized the study, analyzed data, and critically reviewed the manuscript for important intellectual content. All authors approved the final manuscript as submitted and agree to be accountable for all aspects of the work.

## Conflict of Interest

The authors declare that the research was conducted in the absence of any commercial or financial relationships that could be construed as a potential conflict of interest.

## Publisher's Note

All claims expressed in this article are solely those of the authors and do not necessarily represent those of their affiliated organizations, or those of the publisher, the editors and the reviewers. Any product that may be evaluated in this article, or claim that may be made by its manufacturer, is not guaranteed or endorsed by the publisher.
